# Complete Genome Sequence of Universal Bacteriophage Host Strain *Campylobacter jejuni* subsp. *jejuni* PT14

**DOI:** 10.1128/genomeA.00969-13

**Published:** 2013-11-21

**Authors:** Kelly J. Brathwaite, Patcharin Siringan, Joanna Moreton, Ray Wilson, Ian F. Connerton

**Affiliations:** Division of Food Sciences, University of Nottingham, Sutton Bonington Campus, Loughborough, United Kingdoma; Deep Seq, Centre for Genetics and Genomics, University of Nottingham, Queen’s Medical Center, Nottingham, United Kingdomb

## Abstract

*Campylobacter jejuni* strain PT14 is a clinical isolate previously used to propagate bacteriophages in the United Kingdom phage typing scheme. The strain has proven useful in the isolation of *Campylobacter* bacteriophages from several sources, and it functions as a model host in phage therapy experiments with poultry and poultry meat.

## GENOME ANNOUNCEMENT

*Campylobacter jejuni* PT14 has been used to isolate and propagate bacteriophages from environmental samples (1–6). The strain is available from the Public Health England Board as NCTC 12662 (http://www.phe-culturecollections.org.uk). The genome sequence of *C. jejuni* PT14 was determined by pyrosequencing on a 454 GS FLX platform (Roche Diagnostics). A total of 257,645 reads were generated, with an average read length of 352 bases. The reads were *de novo* assembled into a single contig using the CLC Genomics Workbench 6.0. The sequence was compared and confirmed with 5 million 50-bp reads generated using MiSeq technology operating in paired-end mode (Illumina). The genome sequence was annotated using the Prokaryotic Genome Automatic Annotation Pipeline (PGAAP) (7).

The circular genome of *C. jejuni* PT14 was found to be 1,635,252 bp in length, with 1,607 coding sequences and an average G+C content of 30.5%. The genome sequence contains 3 rRNA operons and 41 tRNA genes. No prophage-associated genes or plasmids were found in this genome. *C. jejuni* PT14 contains 26 probable pseudogenes, but notably, it has three annotated pseudogenes from *C. jejuni* strain NCTC 11168, Cj0501, Cj1064, and Cj1470c, which remain intact within the PT14 genome. The clustered regularly interspaced short palindromic repeats and associated genes (CRISPR-Cas) within the genome of *C. jejuni* PT14 were found to be intact, which is an interesting finding considering the sensitive nature of this strain to bacteriophage infections. The CRISPR element is comprised of 32-bp-long spacer sequences and four repeat regions.

*C. jejuni* PT14 was found to contain 27 homopolymeric G+C tracts (defined as containing ≥7 consecutive G+C residues), which bears comparison to the 29 reported in *C. jejuni* NCTC 11168, 25 in RM1221, and 23 in CG8486 (8–11). The locations of the homopolymeric tracts are generally conserved among the *C. jejuni* strains. In *C. jejuni* PT14, there are five tracts residing within intergenic regions, two in probable pseudogenes, and nine within genes of unknown function. Variation in the length of the G+C tracts within genes results in phase-variable expression. Five of the tracts identified in *C. jejuni* PT14, including genes encoding two putative methyltransferases and the invasion protein CipA, show phase variation at the sequence level. *C. jejuni* PT14 also contains a phase-variable A+T region in the gene A911_06060, which encodes a GMP synthase. Phase-variable gene expression has been correlated with modifications of *C. jejuni* surface structures that are required for bacteriophage infection (12). Phase-variable disruption of the Cj1421 pseudogene of *C. jejuni* NCTC 11168 prevents *O*-methyl phosphoramidate attachment to Gal*f*NAc of a capsular polysaccharide, which leads to noninfection by bacteriophage F336 (13) and allows bacteriophage evasion during chicken colonization (14). The Cj1421 homologue of *C. jejuni* PT14 (A911_06918) does not appear in a syntenic region and is not phase variable. However, the Cj1422 homologue (A911_06907), which hinders phage infection by *O*-methyl phosphoamidate attachment to heptose, exhibits phase variation. In summary, although *C. jejuni* PT14 is a phage-sensitive strain, bacterial defense mechanisms still appear to be in place to enable its escape from bacteriophage predation.

### Nucleotide sequence accession number.

The *C. jejuni* PT14 sequence is available under GenBank accession no. CP003871.
